# Rush Medical College

**Published:** 1869-07-15

**Authors:** 


### *Rush Medical College.*

*June 30, 1869.*

EDITOR CHICAGO MEDICAL JOURNAL:

The Spring course for 1869 terminated Wednesday, June 30th. The instructors refer with pride to the character of the class. Twenty young men were enrolled at the beginning of the term, the largest number the spring course has ever attained.

The plan of instruction has given satisfaction. The Clinical advantages of the College Dispensary have been utilized. Over one thousand different cases have presented themselves there for diagnosis and treatment during the year just ended. Aside from this daily practice which the students have seen, the eye and ear infirmary and the County Hospital have presented cases of as great diversity and interest as ever, while the limited number attending the Cliniques favored access to the bedside. A system of training has been introduced, in the course of which each student has had assigned to him a certain number of cases whose history, diagnosis and treatment he was permitted to prepare and conduct under the supervision of the regular attendant.

The term closed in a quiet manner. Most of the students have left the city preparatory to their return at the opening of the regular college session, the 29th of September. We congratulate them. These practical methods of study under the shield of their *Alma Mater*, continued into the long vacation, will tell at the annual examinations.

COM.

East Fairfield, Ohio, *July 2, 1869.*

Prof. Allen:

Dear Sir—The following I send you for your opinion, and if you think it worthy publish it in your Journal:

. Was called to attend a case on Friday, June 25, 1869. The patient, a male about forty-five years of age, was injured in a fight by being struck on the head in the cervical and lumbar regions. Upon examination I found that the injury from which he was suffering was over the fifth and sixth cervical vertebræ. They were thrown forward, the head being thrown backwards. Upon trying to bring the head forward to its natural position respiration would cease. The whole body was paralyzed; respiration very difficult and diaphragmatic. The patient being of full, robust habit, and pulse full and strong, I resorted to the use of the lancet and succeeded in getting about one pint of blood, after which the respiration became a little easier, but in other respects sinking. Called Dr. C. P. Chanlon in consultation; gave as our diagnosis partial displacement of the fifth and sixth cervical vertebræ. About five hours after receiving the injury he commenced coughing, when something snapped, which caused the patient to exclaim "My God, I have broken my neck!" and immediately he began moving his extremities and talking—his head at the same time coming back to its natural position. Improvement rapid. In three days was out of bed, walking about.

Was our diagnosis right or wrong? Please communicate to me your opinion and that of others in regard to this case, as some surgeons deny a recovery after a partial displacement. Yours, respectfully,

J. M. Meloney.

